# The Spectroscopy Village at Diamond Light Source

**DOI:** 10.1107/S1600577518006173

**Published:** 2018-06-01

**Authors:** Sofia Diaz-Moreno, Monica Amboage, Mark Basham, Roberto Boada, Nicolas E. Bricknell, Giannantonio Cibin, Thomas M. Cobb, Jacob Filik, Adam Freeman, Kalotina Geraki, Diego Gianolio, Shusaku Hayama, Konstantin Ignatyev, Luke Keenan, Iuliia Mikulska, J. Frederick W. Mosselmans, James J. Mudd, Stephen A. Parry

**Affiliations:** a Diamond Light Source, Didcot, Oxfordshire OX11 0DE, UK

**Keywords:** X-ray absorption spectroscopy, microfocus spectroscopy, X-ray emission spectroscopy, energy-dispersive EXAFS

## Abstract

This manuscript presents the current status and technical details of the Spectroscopy Village at Diamond Light Source. The Village is formed of four beamlines: I18, B18, I20-Scanning and I20-EDE. The village provides the UK community with local access to a hard X-ray microprobe, a quick-scanning multi-purpose XAS beamline, a high-intensity beamline for X-ray absorption spectroscopy of dilute samples and X-ray emission spectroscopy, and an energy-dispersive extended X-ray absorption fine-structure beamline. The optics of B18, I20-scanning and I20-EDE are detailed; moreover, recent developments on the four beamlines, including new detector hardware and changes in acquisition software, are described.

## Abstract 2

The current status and technical details of the four instruments dedicated to X-ray spectroscopy available at Diamond Light Source are presented.

## 1. Introduction   

The spectroscopy group at Diamond Light Source is formed by the Microfocus Spectroscopy beamline, I18, the Core EXAFS beamline, B18, and the Versatile X-ray Absorption Spectroscopy beamline, I20. The spectrometers were designed to be complementary in the energy ranges that they cover, the X-ray flux they deliver to the sample, and in the spatial and time resolutions that each of them can achieve.

The Microfocus Spectroscopy beamline was built as part of the suite of seven instruments that formed Diamond Phase I (Mosselmans *et al.*, 2009 ▸; Diaz-Moreno *et al.*, 2012 ▸). I18 is an undulator-based beamline that performs spectroscopy experiments in the energy range from 2 to 20.6 keV, with a beam of micrometre size at the sample. This beamline started operation in January 2007, and has been receiving users regularly since then.

The Core EXAFS and the Versatile X-ray Absorption Spectroscopy beamlines were built as part of Diamond Phase II (Dent *et al.*, 2009 ▸; Diaz-Moreno *et al.*, 2012 ▸). The Core EXAFS beamline is focused on functionality and user-friendly capabilities. It is a bending-magnet beamline equipped to perform experiments from the soft to hard X-ray region, from 2 to 34 keV, with a variable spot size ranging from 100 µm to 1 mm. It has the capability to perform Quick-EXAFS (QEXAFS) experiments collecting spectra on the timescale of seconds.

The construction of I20 was a highly complex technical project that involved the construction of two beamlines with two independent wiggler sources in the same straight section (Diaz-Moreno *et al.*, 2009 ▸, 2012 ▸; Diaz-Moreno, 2012 ▸). The first branch, I20-Scanning, is a scanning spectroscopy beamline dedicated to the performance of X-ray absorption spectroscopy (XAS) and X-ray emission spectroscopy (XES) experiments. It received its first users in 2012. The second branch of I20, I20-EDE, is dedicated to energy-dispersive EXAFS (EDE), with the capability of collecting a complete absorption spectrum on timescales ranging from seconds or longer down to microseconds. It started user operation in 2015.

A basic description of each of the beamlines has been given elsewhere, so here will describe the recent improvements and capabilities that have been added.

## 2. The I18 Microfocus Spectroscopy beamline   

The I18 beamline is a hard X-ray microprobe for *K*-edges in the elemental range from phosphorus to molybdenum. Although two experiments have been performed at the technetium *K*-edge, the flux is considerably lower at 21.04 keV than at 20.5 keV, as the cut-off from the rhodium-coated toroid mirror begins to take effect. The beamline uses Kirkpatrick–Baez mirrors to focus the X-ray beam down to spot size of less than 2 µm × 2 µm at the smallest focus, and uses XAS, X-ray fluorescence (XRF) and X-ray diffraction (XRD) as its principal techniques. Since the beamline first started operation, several improvements have been made. Most recently, the standard detector chain and the software for collecting XRF data have been upgraded substantially and the new components are described below.

### 2.1. Detector changes   

The beamline now has two Vortex ME-4 silicon drift detectors (SDDs) (Hitachi High-Technology Science America Inc.) for XRF and XAS measurements. These were purchased seven years apart and the newer one has 1 mm-thick silicon diodes, which gives it comparable efficiency to the previous germanium detector in the higher region of the beamline energy range. This newer detector has the new Cube pre-amplifiers and gives outstanding performance. These are combined with Xspress-3 readout electronics (Quantum Detectors Ltd) to give excellent count-rate performance, both in terms of resolution and linearity.

In particular, the mean FWHM at 1 Mcps (cps = counts s^−1^) per channel is 200 eV on the Mn *K*α line and 300 eV on the Zr *K*α line; furthermore the output can be linearized for XAS data up to 1 Mcps, while for XRF analysis the useable count rate is near 2 Mcps per channel. The older Vortex has a performance approximately half as good as this, so the total count rate capability for XRF of the two-detector setup is around 12 Mcps.

**Figure 1:**
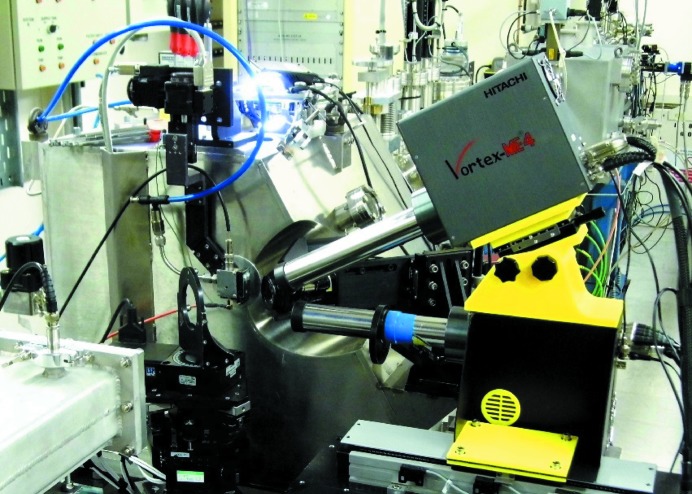
Photograph showing the dual Vortex ME-4 setup on I18.

The detectors are mounted on the same motorized stage (Fig. 1 ▸) with the original beamline Vortex-ME4 detector in the horizontal plane of the beam at 90° to the incident beam, while the other detector sits on top of it at ∼35° to the horizontal, pointing down at the sample. The better count rate performance of this detector enables it to cope with the considerably higher number of elastically scattered X-rays it receives at this angle.

The beamline originally used a Photonic Science VHR camera for XRD. This has now been replaced by a Photonic Science 2×1 array 2k×2k SCMOS camera. This has a substantially faster readout speed than the original camera. It can read out with 1×1 binning at >2 frames s^−1^; this has enabled the development of diffraction tomography, in particular on catalysts under *operando* conditions, on the beamline (*e.g.* Price *et al.*, 2017 ▸).

### 2.2. Software changes   

Diamond beamlines collect data using the *Generic Data Acquisition* (*GDA*) software (Gibbons, 2008 ▸; Rees & Gibbons, 2010 ▸). In 2017, a substantial addition was made to this framework, namely the introduction of the mapping perspective. This perspective is designed to provide a generic tool for the setting up, execution and data visualization from continuous scans using a range of detectors and scan axes on multiple Diamond beamlines. Thus, the software for XRF mapping on the beamline has been completely rewritten.

The architecture of the scan execution involves a triple layer approach. In between the *GDA* user interface, where scan parameters are defined, and the EPICS (Dalesio *et al.*, 1994 ▸) layer, which interfaces with the motor controllers and detectors, sits a middle layer, Malcolm (Cobb *et al.*, 2017 ▸). This implements the high-level configure/run behaviour of the control system components used in continuous scans. Malcolm provides an abstraction layer above EPICS that encloses groups of process variables and presents a higher level scanning interface to *GDA*. This enables it to account for the differences in triggering schemes between different beamlines, leaving *GDA* only having to deal with high-level scan parameters such as motion path and exposure time, rather than requiring to fully understand all the underlying devices. Malcolm was written to interact with the Geobrick system of motor controllers (Delta Tau Data Systems Inc.); hence the motion control system for the sample stages on the beamline was also renewed during the new software implementation.

The process takes advantage of a new circular readout capability of the Xspress-3 system, which has reduced the overhead enormously in continuous XRF scans. For two-way scanning where the data are collected initially with the sample stage moving from left to right and then the next row with the stage moving from right to left, the overhead between rows is just the travel time spent in the acceleration/deceleration zone at the end of each row. In one-way scanning the vast majority of the overhead is the amount of time the scanning stage takes to return to its starting position. This is dependent on the length of the scan.

**Figure 2:**
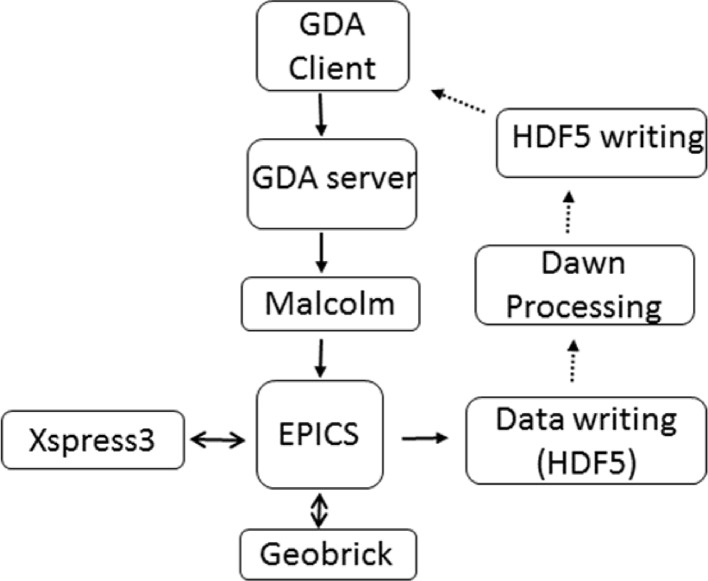
Outline of the process control in the data collection (solid arrows) and data processing and visualization (dotted arrows).

The data collection and the data processing of XRF data are now completely separate tasks (Fig. 2 ▸); the latter is done in real time taking advantage of the HDF5 Single-Writer/Multiple-Reader (SWMR) library. The data are read from the raw output file as it is being written and then passed through a processing routine within the *Dawn* program (Basham *et al.*, 2015 ▸) which executes on a cluster. Fluorescence maps can be constructed either by integrating counts over a range of channels or processed through a *PyMCA* (Sole *et al.*, 2007 ▸) fitting routine if a configuration file has been constructed. The data are then written to a separate SWMR file which is read by the *GDA* client for live display.

For short scans with 20 rows of 3 s duration the overhead per map is around 9%; this is largely the setup time at the start of the map, for longer duration maps it is considerably less. This has enabled the technique of full XANES (X-ray absorption near-edge structure) mapping using fluorescence detection (Brinza *et al.*, 2014 ▸) to be utilized much more extensively. In complex systems, this has the considerable advantage of removing the (unconscious) bias when selecting some points from an XRF map at which to perform spot XANES.

## 3. The B18 Core EXAFS beamline   

B18, the Core EXAFS beamline, collects bending-magnet radiation from Diamond. The 1.4 T dipole provides hard X-rays with a critical energy of 8.4 keV. B18 was opened to users in April 2010, and has now reached almost eight years of operation.

The specifications for B18, coming from the recommendations collected among the UK XAS user community, requested the possibility to cover a wide energy range, from 2.1 to 35 keV, and to provide a focused beam on the sample location to allow for small samples to be investigated. This has put precise constraints on the optical layout. The wide energy range has required the adoption of two independent X-ray mirror configurations, and the use of a double-crystal monochromator equipped with Si(111) for the low-energy range and Si(311) to reach the maximum energy of 35 keV. The optical design (Dent *et al.*, 2009 ▸) developed for B18 takes advantage of the wide aperture of the Diamond standard front-end (3 mrad in the horizontal plane) for bending magnets. As a focusing mirror configuration with both high critical energy (35 keV) and minimal aberrations (in 2:1 geometry) requires toroidal mirrors with short sagittal curvature (38 mm radius for B18), the white-beam fan is split into two quasi-parallel optical paths, corresponding to different metal strips deposited on the first silicon mirror (a 1.2 m-long flat bent unit, collimating the white beam in the vertical direction), coated with chromium and platinum. The focusing device is a monolithic double toroidal mirror, again coated with chromium and platinum, placed downstream of the monochromator, and focusing on the sample position located nominally at 37.5 m from the X-ray source. The double-crystal monochromator (DCM) (Instrument Design Technology) is equipped with Si(111) and Si(311) crystals. The power load is controlled *via* a direct water-cooling system, operating close to room temperature.

One of the essential requirements for the UK community was the ability to provide an efficient scanning mechanism, as it was foreseen that a relevant fraction of assigned beam time would be devoted to *in situ* and *operando* experiments. XAS *in operando* measurements are indeed becoming essential to clarify the mechanisms underlying dynamic processes in chemical reactions of industrial relevance. This request has prompted the development, after the first periods of operation, of a reliable and high-quality continuous scanning system.

At Diamond, fast time-resolved experiments are covered by I20-EDE. Consequently, B18 concentrates on moderate-speed continuous-scanning methods, able to cover the full energy range with minimal interventions in the optical setting. The large angular range needed to reach the low energies ruled out the adoption of fast channel-cut configurations (Müller *et al.*, 2015 ▸; Briois *et al.*, 2016 ▸). We adopted a conventional configuration for the DCM goniometer, allowing for a maximum repetition rate of about 1/3 Hz for EXAFS collection. A UHV-compatible rotary table is driven by a high-speed in-vacuum DC brushless motor, controlled with a Delta-Tau Geobrick LV using a position/velocity feedback loop based on the main DCM Bragg axis encoders. Quadrature signals from four in-vacuum encoder heads placed symmetrically across the main Bragg goniometer are summed to compensate for eccentricity errors. The Bragg motion is optionally synchronized to the second crystal’s gap motor in continuous trajectory scans programmed at low level in the Geobrick controller, to keep constantly a fixed-beam exit. In this regard, no active beam feedback has been necessary up to now. Experience on B18 has shown that the low signal-to-noise associated with the available beam position measurements becomes a source of high-frequency instabilities and increased final noise on experimental data during continuous scans. Irregularities in the parallelism of the second-crystal translation slide are therefore simply compensated using piezo actuators positioned using a look-up table.

The DCM and the triggering/acquisition method can operate bidirectionally on a wide range of angular speeds (from 0.001 to 0.4° s^−1^). This ability is required to enable similar acquisition times for the low/high energy ranges, and to move rapidly to the scan start position when several acquisitions are needed to accumulate significant statistics in fluorescence mode. The mechanism developed for the data acquisition relies on the availability of scalar counters and X-ray fluorescence detector electronics with buffered readout [initially TFG2 and Xspress-2 (Farrow *et al.*, 1995 ▸)] and a programmable encoder-based hardware triggering system, Zebra (Cobb *et al.*, 2013 ▸).

Our experience has shown that the continuous-scanning method brings substantial advantages over the traditional approach, so this is now the default acquisition method for all XAS experiments. This includes not only the case of time-resolved *in operando* experiments but also acquisitions on dilute systems.

(i) The continuous scan improves systematically the overall acquisition rate, by removing the dead-time between the collection of individual experimental data points needed in conventional step-scan mechanisms.

(ii) At the same time, the mechanics is not subject to intermittent acceleration and deceleration cycles, most likely reducing the wear of the critical motion components. B18 has accumulated to date over 300000 energy scans, with minimal interventions on the main scanning components.

(iii) The overall data quality is not affected by the continuous motion, where appropriate strategies are adopted. Where time resolution is not relevant, speed is usually adjusted so acquisitions are run over a full EXAFS scan for a total duration of 1 to 3 min. Given the hardware buffer for Xspress-2 is limited by a maximum number of 4000 frames, the typical acquisition time per experimental point is of the order of 40 ms. For transmission experiments the adoption of V2F100 high-speed voltage-to-frequency converters guarantees that the acquisition noise is not dominated by acquisition counting statistics for even the fastest rates; an analysis of the final signal-to-noise on transmission experiments, as a function of the total acquisition time, has shown indeed a power-law behaviour very close to what is expected from photon-limited statistics (Dent *et al.*, 2013 ▸).

(iv) Quick scanning allows for a rapid assessment of the data quality during the acquisition setup phase. Having the possibility to collect a preliminary spectrum in a few seconds allows the users to identify potential issues, over the full experimental range. This goes beyond the evaluation of, for example, the signal intensity at different sample locations, which can be obtained conventionally by scanning the sample positioners at a few energies selected across the absorption edge. Full scans allow the identification of non-compensated monochromator glitches, often symptomatic in transmission acquisition of sample areas affected by pinhole or inhomogeneous materials distribution. Also they allow the optimization of fluorescence measurements affected by the presence of diffraction peaks from crystalline materials. This is a well known and common challenge for XAS experiments, and affects the acquisition on thin films deposited on crystalline substrates, single crystals and dopants in crystalline systems prepared in powder form. Rapid scanning allows mapping the data quality *versus* sample position, angular alignment and detector locations with high efficiency.

(v) It is possible to collect full high-quality XAS spatial maps on samples intrinsically characterized by inhomogeneous component distributions. This is the case in art and archaeology, corrosion science and palaeontology experiments where a micro-focus beam is not always necessary.

(vi) It is possible to minimize the effects of radiation damage on sensitive samples, by collecting several repeated scans at different locations.

The overall efficiency given by a reliable optical configuration and the continuous scanning mechanism, coupled to the availability of multi-element fluorescence and XRD detectors, has allowed B18 to develop into a high-quality high-throughput EXAFS instrument. In particular, it has made it possible, in addition to the direct access and long term proposal modes available at Diamond, for the development of rapid access and the beamline access group (BAG) modes. In the first case, experiments with a limited number of samples and minimal sample environment requirements, with a justification for urgency, can be programmed with a lead time substantially shorter than the usual six-months beam time allocation cycles allow. The rapid access experiments are usually run by the beamline team, scheduled in automated nightly acquisitions following short direct-access experiments or commissioning beam time days. The BAG mode gives periodic access to a consortium of institutions collaborating on the scientific, technical and logistics levels, with interest focusing on common areas. On B18, BAG days involve the rapid succession of individual experiments co-ordinated usually by an expert users’ core team, able to drive independently the data collection sessions of new groups and in­experienced researchers, supported by the beamline staff for major beamline configuration setup changes.

**Figure 3:**
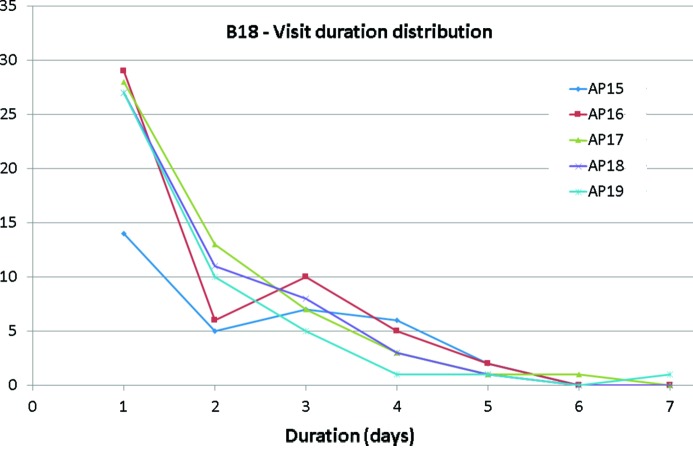
Experimental visit duration distribution on B18 for recent six-month beam time allocation periods.

With the mentioned developments, the number of experiments scheduled on B18 has steadily increased, opening access and encouraging the growth of a diverse XAS user community. Fig. 3 ▸ reports the duration distribution of experiments on B18 on five recent allocation periods, and indicates that one-day experiments (including rapid access, BAG and direct access mode proposals) are the most common route. This indicates that XAS is now an essential tool for investigations in the areas mostly covered by B18 (catalysis, energy materials, environmental sciences), and that further steps towards full automation (Figueroa *et al.*, 2018 ▸) and data analysis procedures are necessary to support the increasing demands for beam time access of the XAS user community.

## 4. The I20 Versatile X-ray Absorption Spectroscopy beamline   

**Figure 4:**
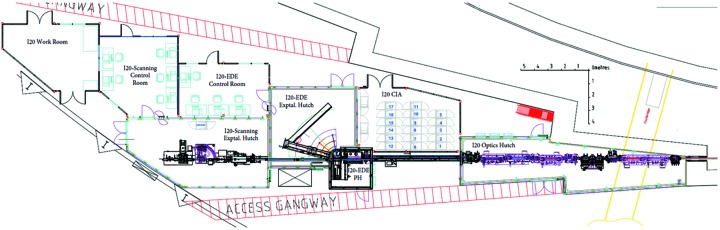
Plan layout of I20 showing the different hutches and control cabins for both branches of the beamline.

Beamline I20 covers scientific applications in several disciplines, from physics, chemistry and biology through materials science and the environmental and geological sciences. To cater to all these communities, the beamline has been divided into two independent spectrometers that can operate simultaneously, where each spectrometer is optimized for different types of experiments. As previously described in the *Introduction* [Sec sec1], the first branch of the beamline is a scanning X-ray absorption and emission spectrometer, that is focused on the delivery of an X-ray beam with high flux and high spectral purity. The second branch works in a dispersive configuration optimized for time-resolved XAS experiments. A schematic of the layout of the two branches of I20 is shown in Fig. 4 ▸.

Each branch of the I20 beamline takes its radiation from a dedicated insertion device, but these two X-ray sources share the same straight section in the ring. These insertion devices are two *ex vacuum* wigglers that deliver the X-ray beams to the two branches. Each wiggler is a hybrid device of 83 mm period. The wiggler for the scanning branch is 2 m in length and the wiggler for the EDE branch is 0.7 m long, due to the space constraints imposed by the length of the synchrotron straight. The minimum gap for the wiggler supplying photons to the scanning branch is 11 mm, whilst the EDE wiggler nominal gap is 18.5 mm. The capability of changing the gap of the insertion device is implemented on both devices, so when working at low energies the gap of the wigglers can be opened to reduce the emission of high-energy photons, and at the same time reduce the total heat load imposed on the optical elements.

The full separation of the two different photon beams to allow the simultaneous operation of the two branches is achieved by adopting a canted configuration of the two insertion devices. The wiggler for the scanning branch is located 0.75 m away from the centre of the 5 m straight section of I20, and canted 0.8 mrad outwards from the storage ring with respect to the machine centre line. The wiggler for the dispersive branch is located 1.856 m downstream of the first device, and in this case is canted inwards by 2.9 mrad towards the storage ring. This configuration gives a total separation angle of the two photon beams of 3.7 mrad, measured from the centre of their emissions. The proximity of the two beams has imposed very strict demands on the design of the optical elements of the two branches of I20 and, as a result, many of the components of the beamlines have been designed in-house.

Due to the proximity of the two beams, the two beamlines share the same vacuum space in the optics hutch. This vacuum space is separated from the machine vacuum by two 300 µm cooled windows; a CVD diamond window in the case of the scanning branch and a beryllium window in the case of the EDE branch. The two branches are fully independent after the beams exit the optic hutch when the separation between the two beams is large enough to facilitate delivery into different experimental hutches.

In the following sections the main characteristics of the two branches of the beamline are described. While the focus is on the improvements and capabilities that have been added in recent years, a brief description of the optical elements of each of the branches is also given. More information about the optical design of the two beamlines are given by Diaz-Moreno *et al.* (2009 ▸).

### 4.1. I20-Scanning branch   

The scanning branch of I20 is particularly well suited for the determination of the local structure around a photoabsorbing atomic site that is present at low concentration and/or in X-ray unfavourable heavy-element matrices. The beamline covers the energy range from 4 to 20 keV when this mode of operation is selected, although it is expected to extend the energy range up to 34 keV in the future.

I20-Scanning also includes a second end-station that is built around an X-ray emission spectrometer, operating in the energy range from 4 to 20 keV covering the *K*-edges of the first row and the *L*-edges of the third row of the transition metals. This instrument is regularly used to perform high-energy-resolution fluorescence detection (HERFD) experiments, as well as non-resonant and resonant X-ray emission spectroscopies (XES and RXES).

**Figure 5:**
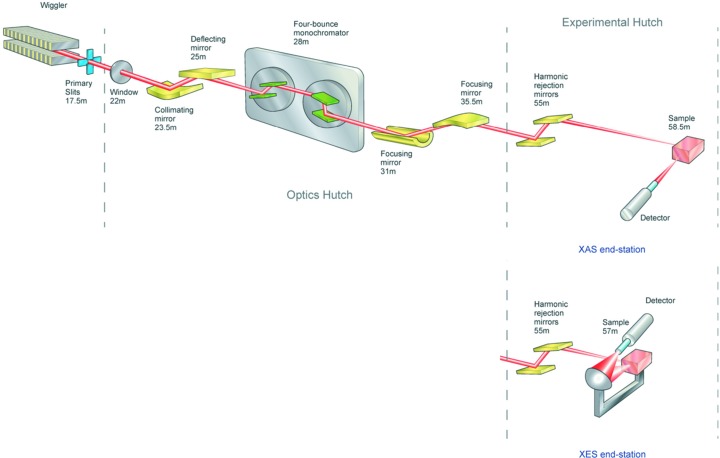
Optical layout of the I20-Scanning branch, showing the two different end-stations.

A schematic layout of the scanning branch of the beamline is shown in Fig. 5 ▸.

#### 4.1.1. I20-Scanning optical design   

The main optical element of the scanning branch is the four-bounce monochromator, necessary to fulfil the strict requirements of the beamline regarding stability and spectral purity (Sutter *et al.*, 2008 ▸; Duller *et al.*, 2008 ▸, 2012 ▸). The device has been designed and built in-house, and is based on two synchronized counter-rotating axes, each one carrying a pair of crystals in a traditional double-crystal configuration. The two axes are set in a dispersive configuration. This device configuration means that the spectral purity of the monochromatic beam depends solely on the crystal cut used, and is unaffected by other factors such as the divergence of the incident beam, angular movements of the source or changes in heat load on the first monochromator crystal. This consequently provides an unprecedented level of stability in the energy calibration and resolution achieved by the beamline. The monochromator is equipped with cryogenically cooled Si(111) crystals, covering an energy range from 4 to 20 keV. In future the use of Si(311) crystals is foreseen to extend the energy range to 34 keV.

A pair of water-cooled vertically deflecting mirrors are placed upstream of the monochromator to condition the beam. Both mirrors operate at 2.3 mrad incident angle, and are coated with two different stripes, one rhodium and one platinum. The first mirror is an upwards-deflecting collimating mirror, that is critical for maximizing the flux throughput of the monochromator. The second is a flat downwards-deflecting mirror whose function is to restore the horizontal trajectory of the beam in order to simplify the alignment of the monochromator.

Downstream of the monochromator two more vertically deflecting mirrors can be found. Both these mirrors also work at a nominal incidence angle of 2.3 mrad. The first mirror is an upwards-deflecting horizontally focusing mirror of cylindrical geometry. The second mirror is a vertical focusing mirror. Both mirrors have been designed to focus the X-ray beam at the two different sample positions in the experimental hutch. The focal distance on the horizontal focusing mirror is changed by changing the incidence angle, whilst the vertical focusing mirror focal length is changed by varying the radius of curvature.

A set of flat vertical-deflecting mirrors placed in the experimental hutch are used to remove higher harmonics from the monochromatic beam when working below 18 keV. The mirrors have two stripes for optimal performance, rhodium and silicon, and the mirror pair operates at angles between 3.5 mrad and 5 mrad to ensure the rejection of higher harmonics over its entire energy range of operation.

#### 4.1.2. I20-Scanning experimental station   

The monochromated and focused X-ray beam is then transferred to the experimental hutch of I20, housing two different end-stations, each one equipped with its own detectors. The size (FWHM) of the focal spot at each of the sample positions is 400 µm × 400 µm (H × V).

**Figure 6:**
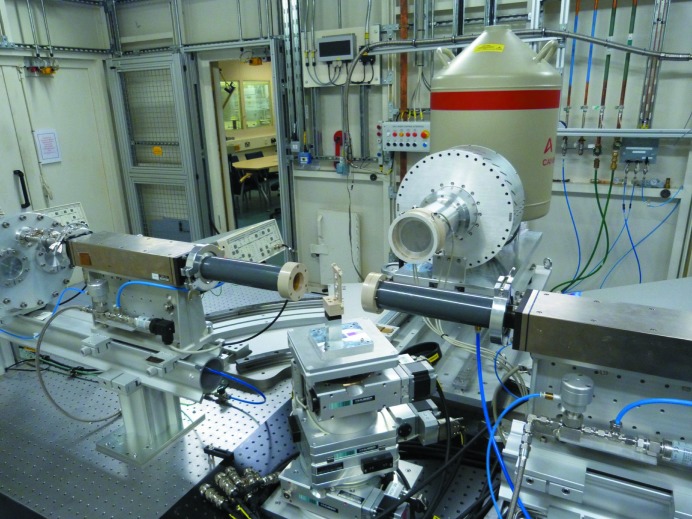
View of the X-ray absorption spectroscopy end-station in the I20-Scanning branch.

The first end-station is dedicated to performing conventional XAS experiments, both in transmission and fluorescence detection modes. A large optical table is used to support the detectors and associated electronics as well as the sample environment available in the beamline (Fig. 6 ▸).

Three OKEN 30 cm-long ion chambers are used to perform transmission measurements by measuring the incident and transmitted intensity of the X-ray beam. An automatic gas-filling system fills the ion chambers with the optimized gas mixture (helium plus argon, nitro­gen or krypton) for 20% and 80% absorption in *I*_0_ and *I*_t_ (*i.e.* the intensity of the incident and transmitted radiation, respectively), respectively, for the entire operational energy range of the beamline. The current measured by the ion chambers is amplified by a fast amplifier (SR570 Stanford Research System) and the output converted by a 1 MHz voltage-to-frequency converter (NOVA N101VTF). For those experiments performed in fluorescence detection mode, a 64-element monolithic germanium solid state detector (Canberra) is available (Chatterji *et al.*, 2016 ▸). The detector sits on a large rotational stage that allows it to be located at different angles with respect to the incident X-ray beam, ranging from −20° to +25° with respect to the zero position equal to the detector being perpendicular to the X-ray beam. This allows the study of samples that are in sample environments that make it difficult to access the fluorescence emission. The readout of the multi-element detector uses the Xspress-2 adaptive event filter system, developed at STFC as part of Xspress (Farrow *et al.*, 1995 ▸). This system delivers an increase of the raw count rate capability of the detector in comparison with other commercially available data acquisition electronics. This system is now being upgraded to Xspress-4, developed at Diamond, a new Digital Pulse Processor (DPP) hardware and firmware architectures developed to fully implement cross-talk corrections between elements in monolithic detectors in real time. For operation at low energy, a Vortex-ME4 four-element SDD (Hitachi High-Technology Science America Inc.) is available. This detector is used with the compact Xspress-3 readout (Quantum Detectors Ltd), based on the STFC Xspress algorithm and optimized for operation with SDDs.

Commercial and in-house-developed sample environments are available at the beamline: cryogenic helium and nitro­gen cryostats, a liquid-nitro­gen cryostream, furnaces designed to be used in the fluorescence detection experimental geometry, plug-flow reactor cells, *etc.* All these different experimental setups are placed on a dismountable breadboard of dimensions 600 mm × 600 mm, that is placed on the experimental table with high reproducibility. This reduces the downtime needed for changing experimental setups on the beamline, as the sample environment can be pre-aligned and assembled offline before the experiment is due to start.

**Figure 7:**
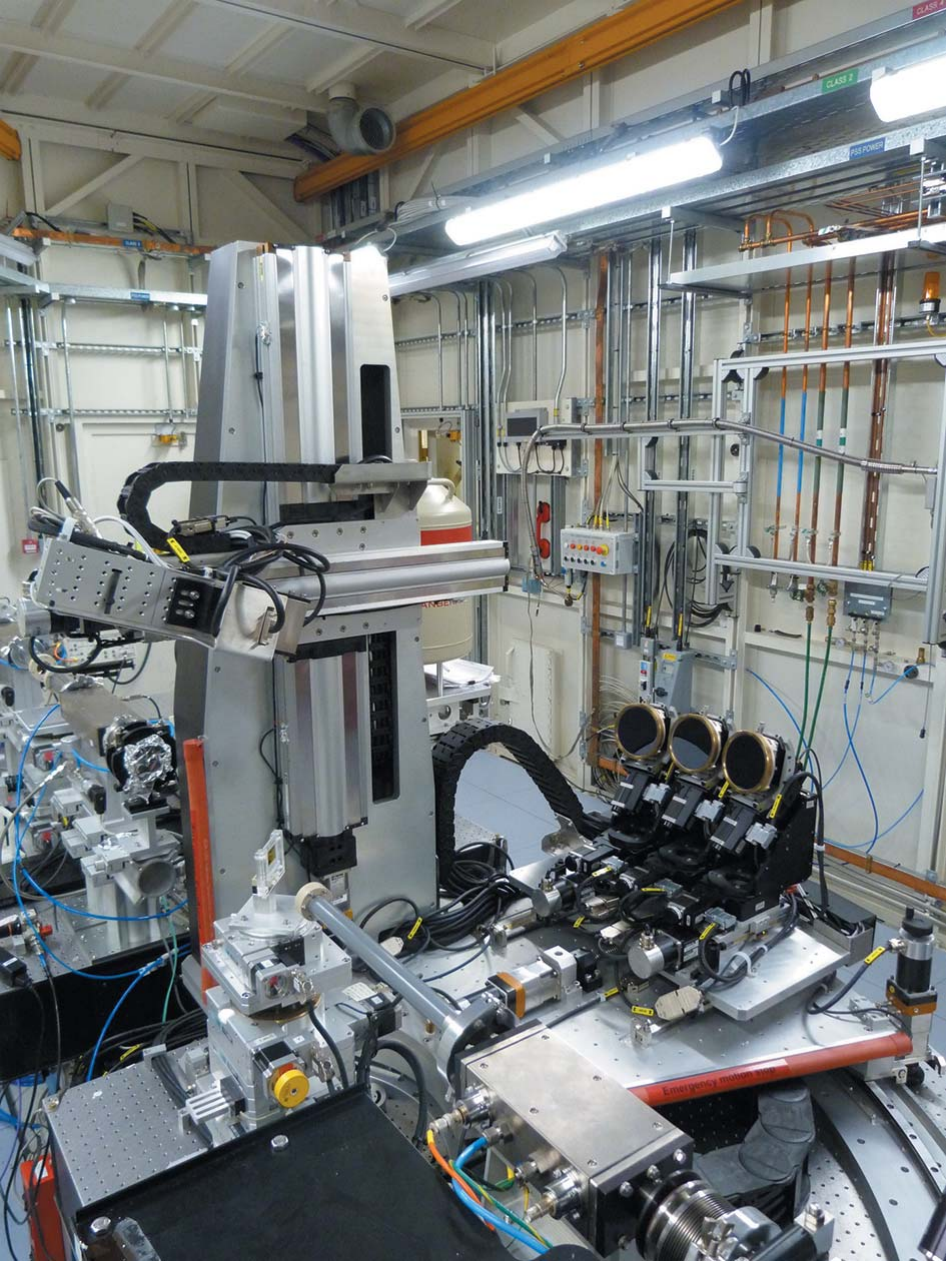
View of the X-ray emission spectrometer installed in the I20-Scanning branch.

The X-ray emission spectrometer is based on a 1 m-diameter Rowland circle, that operates in the Johann configuration in the vertical plane (Fig. 7 ▸) (Johann, 1931 ▸). The spectrometer is usually placed perpendicular to the incident X-ray beam, although it can be rotated from −10° to +59° around the nominal position. It is equipped with three spherically bent analyser crystals of 100 mm diameter, each covering 0.0025π steradian. In order to increase the signal collected, allowance has been made in the design for a future upgrade to a five-crystal system. The spectrometer works at angles close to backscattering, from 70° to 85° to achieve good energy resolution for the operational energy range. A large suite of silicon analyser crystals (XRS TECH LLC) (3 crystals × 8 different crystal cuts) and germanium analyser crystals (XRS TECH LLC) (3 crystals × 3 different crystal cuts) are available at the beamline in order to cover the *K*α and *K*β emission lines of the first row of the transition metals.

**Figure 8:**
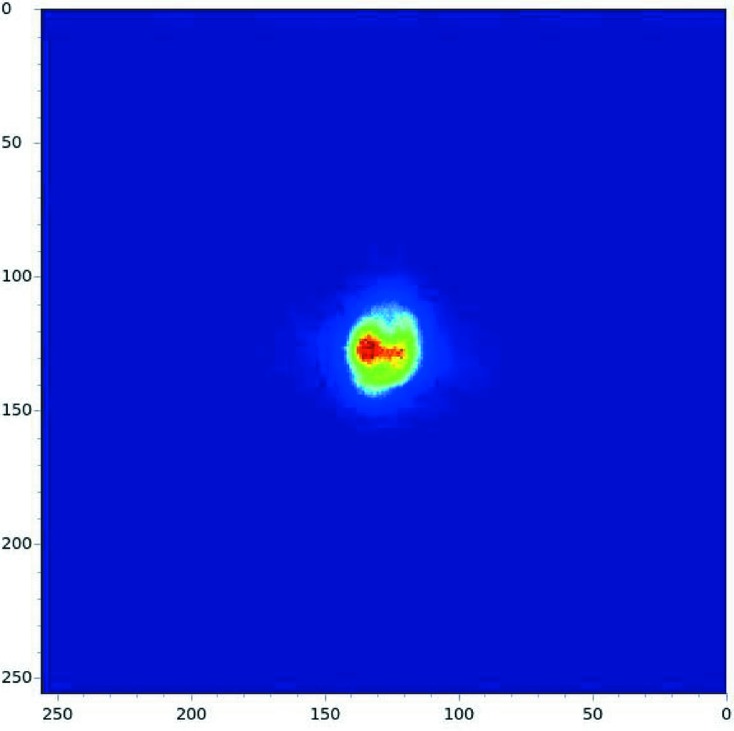
Image of the X-ray beam at the Medipix detector using the emission spectrometer. The image has been taken at the Zn *K*β emission (9572 eV) using one Ge(555) strip-analyzer crystal operating at 82.44°.

Although several detectors are available for use with the spectrometer [Vortex-ME1 one-element SDD (Hitachi High-Technology Science America Inc.) for low-concentration samples, an avalanche photodiode for concentrated samples], it usually operates with a pixel-area detector (one-element Medipix) capable of imaging the focus achieved by each analyzer crystal, reducing the alignment time of the spectrometer significantly (Fig. 8 ▸) (Plackett *et al.*, 2013 ▸). The one-element Medipix detector is based on a 256 × 256 pixels Medipix3 chip, with a 55 µm pixel size, and it is used with the Merlin readout electronics. Recently, the detector has been upgraded to a four-element Medipix system (256 × 1024 pixels) maintaining the same read-out electronics, to further improve the efficiency of the spectrometer.

For operation at low energy a helium enclosure has been added to the emission spectrometer to minimize the air path that the X-ray beam needs to go through. The enclosure has been built with flexible material so it can accommodate the range of movement of the analyzer crystals when the spectrometer is scanned across an emission line. Two large Kapton windows have been added to the enclosure, one for the analyser crystals and one for the detector.

Due to the backscattering geometry that the spectrometer operates at and the presence of the helium enclosure, the space available for experimental equipment at the sample position is very limited. The sample environments available at the beamline for the XAS end-station do not fit in the space in the spectrometer, so specially designed in-house or adapted sample environments have been incorporated. At the current time, these sample environment setups include a Linkam stage that is available to work in the temperature range from −196 to 600°C, and an in-house-designed plug-flow reactor to allow the study of samples at high temperature under gas flow.

### 4.2. I20-EDE branchline   

**Figure 9:**
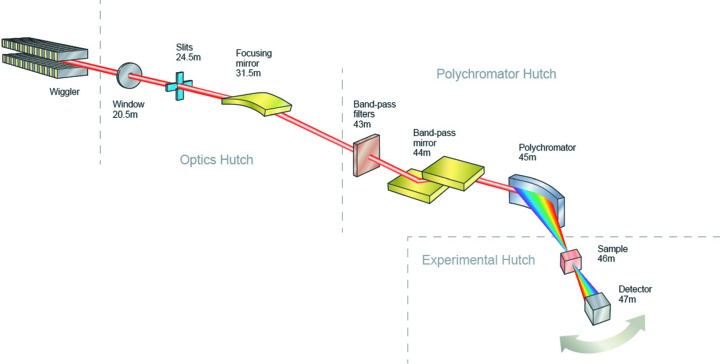
Optical layout of the I20-EDE branch.

The second branch of I20 is devoted to the performance of EXAFS spectroscopy experiments in the dispersive geometry, over the energy range from 6 to 26 keV. This allows the spectrometer to access the absorption edges of most of the periodic table, *via* the *K*- or the *L*-edges. The unique configuration of the I20-EDE branch is particularly well suited for *in situ* and *operando* time-resolved studies of chemical reactions. The system allows the spectrometer to follow the structure of the absorbing element over time scales ranging from seconds down to milliseconds or even microseconds. In the dispersive configuration, a curved crystal is used to spatially separate the energies of a polychromatic beam, that is then coupled with a position-sensitive detector to allow the whole energy spectrum to be collected in a single shot. This combination allows the spectrometer to achieve very short data collection times. Scientifically, this branch of I20 is particularly suitable for the study of catalytic processes, both in heterogeneous and in homogeneous media. An added advantage of the dispersive configuration is the small size of the X-ray beam focus achieved at the sample position. This characteristic, together with the absence of any mechanical movement of the optical components of the beamline when collecting data, makes this beamline ideal for the study of samples at high pressure with diamond anvil cells, or systems where the spatial resolution is important. The schematic layout of the dispersive branch of the beamline is shown in Fig. 9 ▸.

Two main criteria have been followed when designing this branch. First of all, a broad energy bandpass is necessary to allow an extended energy range of the X-ray absorption spectrum to be collected. The bandpass achieved in I20-EDE is 10% of the working energy at all energies. In addition, a high flux on the sample is needed to facilitate the performance of experiments with a high time resolution. These two criteria are fulfilled by using a wiggler as the X-ray source and a large polychromator crystal.

#### 4.2.1. I20-EDE optical design   

The first optical element of the beamline is the primary slits placed in the optics hutch. These slits define the size of the beam in the horizontal and vertical directions. The nominal vertical size is equal to 0.12 mrad, and it is kept constant over the entire energy range. However, the horizontal size of the beam is set by the energy at which the beamline is operating, ranging from 1.6 mrad for low-energy operation down to 0.8 mrad for the high-energy limit. This is necessary in order to illuminate the entire optical length of the polychromator crystal (250 mm) at all energies, providing the large energy band-pass necessary to collect a full EXAFS spectrum.

Vertical focusing at the sample position is achieved with a water-cooled downwards-deflecting mirror downstream of the slits. The mirror operates at 3 mrad incident angle, and is coated with two stripes, rhodium and platinum, in order to cover the operating energy range of the beamline.

Housed in a separate hutch is the polychromator, the main optical component of the beamline. It consists of a single-bounce curved crystal working in the horizontal plane (Sutter *et al.*, 2010 ▸). The polychromator operates in a Bragg geometry. The curvature of the 320 mm-long silicon blade is dynamically changed for different working energies in order to obtain the required ellipse. To achieve this, the crystal is mounted in a four-point bender, and the essential cooling of the crystal is achieved by immersing part of it in a water-cooled copper bath filled with InGa eutectic. The whole assembly operates under a helium atmosphere, and a series of interlocks are in place to ensure that there is always a slight overpressure of helium inside the vessel. Two different crystal cuts are used in order to cover the operational range of the beamline. The Si(111) crystal cut is used to cover the energy range from 6 to 12 keV while the Si(311) covers the range from 7 to 26 keV. The change of crystal requires opening the vessel that contains the crystal and the bender.

The main drawback of working in the Bragg configuration is the large penetration of the X-rays at higher energies, compromising the energy resolution that is achieved (Hagelstein *et al.*, 1995 ▸). This effect also significantly degrades the quality of the focal spot achieved at the sample position, as large tails appear in the horizontal plane. To mitigate this problem, a polychromator working in the Laue configuration is being considered as a future upgrade to improve the beamline’s high-energy capabilities.

Two other optical components are located upstream of the polychromator inside the same hutch: a set of two water-cooled mirrors and three actuators containing different thickness pyrolytic carbon filters (Advanced Carbon Technologies, Inc.). A combination of filters and mirrors is used to reduce the heat load on the polychromator, keeping it below 80 W, to avoid thermal effects that degrade the quality of the X-ray beam. The mirrors are also used to reduce the harmonic content in the X-ray beam in the energy range from 6 to 18 keV. The first mirror is upward deflecting while the second mirror is downward deflecting. They have two stripes, silicon and rhodium, and operate at incident angles ranging from 3 to 5 mrad. To simplify the mechanism, the second mirror is large enough to allow the beam to walk for different angular settings.

Just downstream of the mirrors but upstream of the polychromator are a set of vertical and horizontal water-cooled slits. These slits are needed to clean the main beam from scattering from the mirror. A 500 µm beryllium window is installed downstream of the slits, to separate the vacuum section of the beamline from the polychromator helium enclosure.

After the polychromator the X-ray beam travels in air to reach the experimental hutch.

#### 4.2.2. I20-EDE experimental station   

As mentioned above, one of the main activities of the dispersive branch of I20 is the study of reactions *in situ* and under *operando* conditions. The typical experiment performed on this beamline uses additional instrumentation that generally has to be placed around the sample, such as gas distribution devices, plug flow reactors, flow cells with hydro­static pumps, the necessary electronics to synchronized experiments, *etc*. and frequent use of simultaneous complementary techniques such as mass spectrometry, UV–Vis, IR or Raman spectroscopies is requested. The experimental hutch of I20-EDE is relatively large to allow all this instrumentation to be located around the sample. To facilitate ease of operation, the hutch is based on a modular setup, catering not only for the different sample environments used in the beamline but also different detectors.

**Figure 10:**
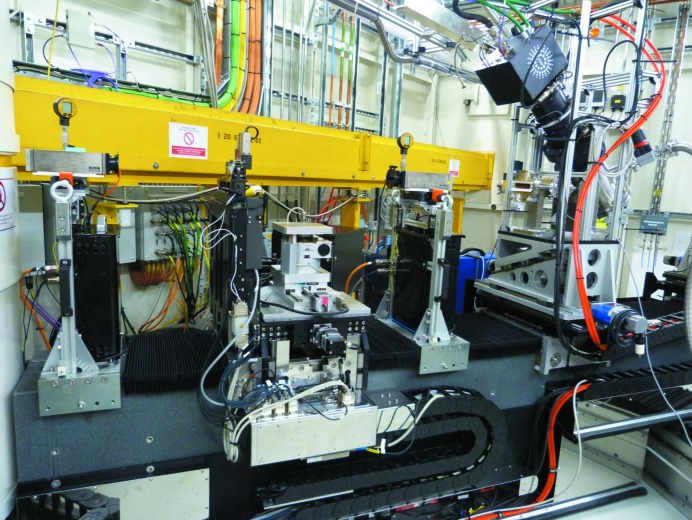
View of the I20-EDE end-station.

The end-station of the EDE branch houses a 3.5 m movable granite arm that rotates to twice the angle selected by the single-bounce crystal polychromator to select the working energy (Fig. 10 ▸). At one extreme, the granite block rests on a hemispherical air-bearing placed under the polychromator and rotating around the same vertical axis to maintain alignment. At the other end, the movable arm rests on another fixed granite beam, 4.5 m long. Between the two granite beams, another air-bearing is found. When the arm needs to be moved, the air bearings are engaged, and the rotating arm travels on top of the fixed arm. When collecting data, the air is switched off and the granite beam is kept in position by its own weight, reducing the vibrations transmitted by the experimental hall floor.

On the movable granite beam, and moving along the path of the X-ray beam, several stages are placed and these are also moved using air bearings. These stages support the different detectors and sample environments, and follow the same principle of operation as the main granite arm: the air bearing is in operation when the equipment is being aligned to the X-ray beam, and the stages are kept stable by their own weight when the air is switched off.

One of these stages holds the different sample environments available at the beamline. As some of this equipment is quite bulky, this stage has a vertical and a horizontal translation, so only stages with small footprint are used if additional fine alignment is needed. In addition, a second stage is used to help focusing the X-ray beam at the sample position. This stage is also built on an air-bearing and when in use is attached to the sample stage. It contains an X-ray camera and a set of vertical slits to optimize the horizontal focus generated by the polychromator. It also contains a foil holder, in order to calibrate the energy of the beamline. The stage also houses a laser pointer aligned in the vertical plane to the X-ray beam to ease the alignment of the sample and detectors.

Two main data collection modes are available in the dispersive branch of I20.

As previously mentioned, the dispersive data collection mode uses a position-sensitive detector to collect the entire energy spectrum in a single shot. This operation mode has the advantage of the parallel acquisition of the data, but in order to maintain the relationship position-energy the experiments need to be performed in transmission detection mode, and this restricts the lower concentration limit that the beamline can achieve. Two detectors are available at the beamline: a FReLoN CCD camera (ESRF) (Labiche *et al.*, 2007 ▸) is used for following reactions in the second to millisecond timescales and a 1024 pixel microstrip germanium detector is also available for studying faster microsecond processes. The microstrip detector is able to collect data in 1 µs with a readout time of 10 µs (Salvini *et al.*, 2005 ▸).

The turbo-XAS method for data collection with the dispersive configuration, originally developed at the ESRF (Pascarelli *et al.*, 1999 ▸), has also been implemented at the beamline. In this mode, a monochromatic beam is obtained by scanning a narrow horizontal slit (turbo-slits) through the energy-dispersed fan of radiation immediately after the polychromator. The sequential data acquisition has the advantage of allowing the simultaneous recording of the intensity of the incident and transmitted radiation (*I*_0_ and *I*_t_), and makes it possible to use the fluorescence detection method. In the I20-EDE branchline, the turbo-slits assembly is located at 335 mm from the centre of rotation of the polychromator, on a large motorized arc. When collecting data, the turbo-slits are placed in the position on the arc that corresponds to the working energy. The slits themselves are located on an air bearing and are able to scan at a maximum speed of 200 mm s^−1^. The slits are scanned in a continuous manner at a constant velocity, typically around 50 mm s^−1^. Position-based triggering of the detectors is achieved *via* a Zebra unit (Quantum Detectors Ltd). The total time per scan is determined by the speed chosen for the slit scan, that itself mainly depends on the sample concentration, and the total range that the slits need to scan. This in turn depends on the size of the fan. As an example, the typical fan size at the slit position for 7 keV operation using the Si(111) monochromator crystal is 50 mm, while the size is only 30 mm when working at 12 keV under the same conditions. The total scan range can also be limited by the type of experiment being performed; the time per scan is reduced if only a short scan is collected. Currently the data collection is unidirectional, which results in a small dead-time between scans. The dead-time is determined for every experiment as it depends on the length of the collected scan, although is typically of the order of 0.2 s.

**Figure 11:**
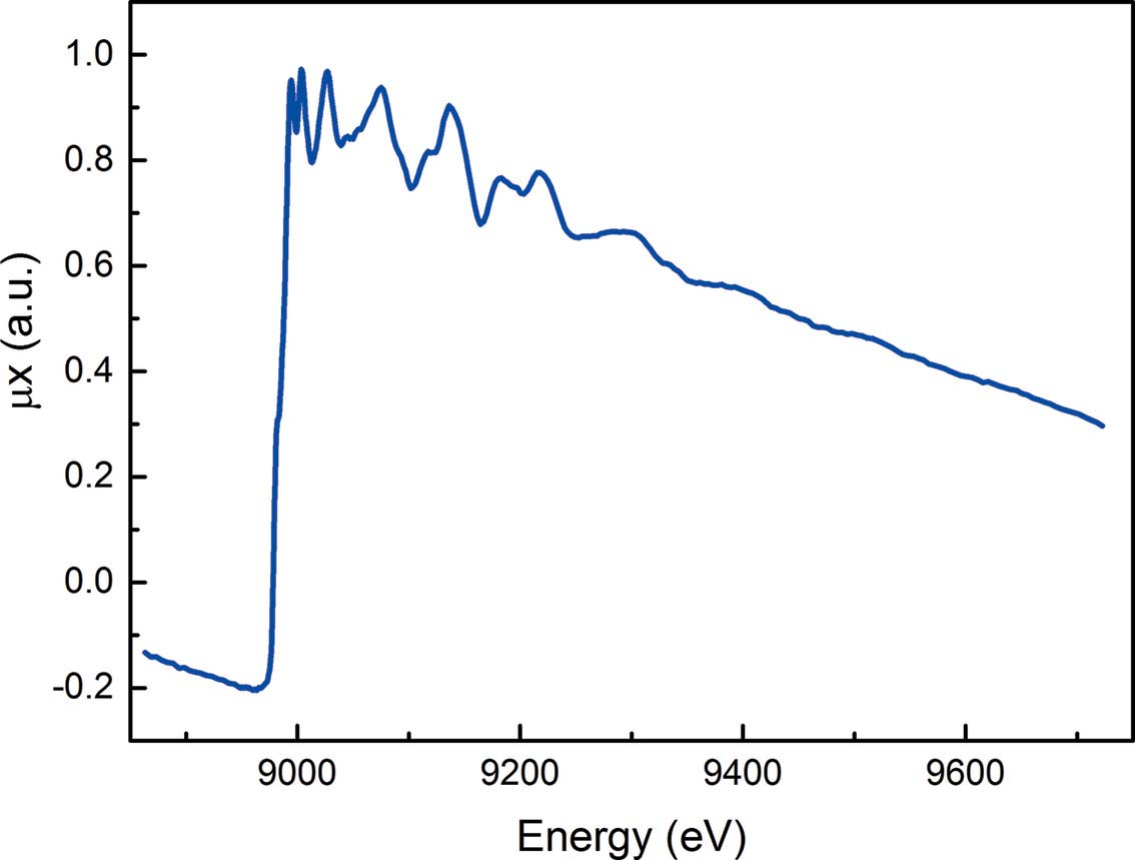
Cu *K*-edge absorption spectrum of a copper foil collected using the turbo-XAS acquisition mode. The spectrum was collected in 1 s.

For transmission detection mode, two 15 cm OKEN ion chambers are used. The ion chambers are set on two independent air-bearing stages, placed on the movable granite beam. The current generated by the ion chambers is amplified by fast current amplifiers (SR570 Stanford Research System) that are also placed in the same air-bearing stages. The output signal is converted by a voltage-to-frequency converter (V2F100 Quantum Detectors Ltd) working at 25 MHz. A typical scan of a copper foil measured using the turbo-XAS in transmission mode is shown in Fig. 11 ▸.

When using the fluorescence detection mode, a Vortex-ME4 four-element SDD is used to measure the radiation emitted by the sample, together with the Xspress-3 read-out electronics.

## 5. Concluding remarks   

The Diamond Spectroscopy Village is formed by four fully operational beamlines. The beamlines are complementary in many aspects, such as the energy range they cover, the focal size they achieve or the time resolution they are able to reach. Each beamline has its unique characteristics and they are optimized for performing different types of experimental studies. The unique characteristics of the four beamlines described in this paper allows the Spectroscopy Village to support a science programme over many different scientific disciplines, from chemistry and catalysis to environmental science, materials science, physics, biology, medicine and cultural heritage.

The success of this approach is reflected in the large number of experimental visits that the village hosted each year, and the number of publications produced. This is particularly the case for the more mature beamlines in the village, I18 and B18. I18 hosted 40 and 49 peer reviewed user experiments in 2016 and 2017, respectively, while B18 hosted 73 and 95 during the same two years. During the same period, I18 produced 62 peer reviewed papers and B18 103 papers. Due to the complexity of bringing the two independent operating branches of I20 into full operation, this facility is still ramping up its activity and output.

The beamlines share many common features, in order to make their operation easier for both the user community and the Diamond teams that support them. The majority of the beamline components are controlled using EPICS (Dalesio *et al.*, 1994 ▸); and the user interface is provided by the *GDA* program (Gibbons, 2008 ▸; Rees & Gibbons, 2010 ▸). The basic GUI interface for the four beamlines is similar, although each beamline has specific GUIs for the unique characteristics of the beamlines: an XRF mapping GUI has been developed for I18, B18 uses an application written to run the QEXAFS experiments and I20 has specific control software to run the emission spectrometer.

The beamlines share the usage of three preparation laboratories placed in the peripheral areas of the experimental hall, close to the beamlines. These facilities are available for users and staff of the Spectroscopy Village for sample preparation.

The development of the beamlines is continuing. Many improvements in data acquisition and analysis are on-going to make the beamlines simple to operate for both expert and non-expert user groups. As the experiments are becoming increasingly more complex, new instrumentation and sample environments are being developed to increase the versatility of the beamlines.
